# A new and unusual species of *Amastigogonus* Brölemann, 1913 from Tasmania, Australia (Diplopoda, Spirostreptida, Iulomorphidae)

**DOI:** 10.3897/zookeys.687.14872

**Published:** 2017-08-01

**Authors:** Robert Mesibov

**Affiliations:** 1 West Ulverstone, Tasmania 7315, Australia

**Keywords:** Millipede, gonopod

## Abstract

*Amastigogonus insularis* **sp. n.** is described from Schouten and Tasman Islands off the east coast of Tasmania, and a key is presented for the identification of males of Tasmanian *Amastigogonus* species. The new species differs from the 10 previously described species of *Amastigogonus* in having a reduced coxite process on the anterior gonopod.

## Introduction

**Figure 1.**
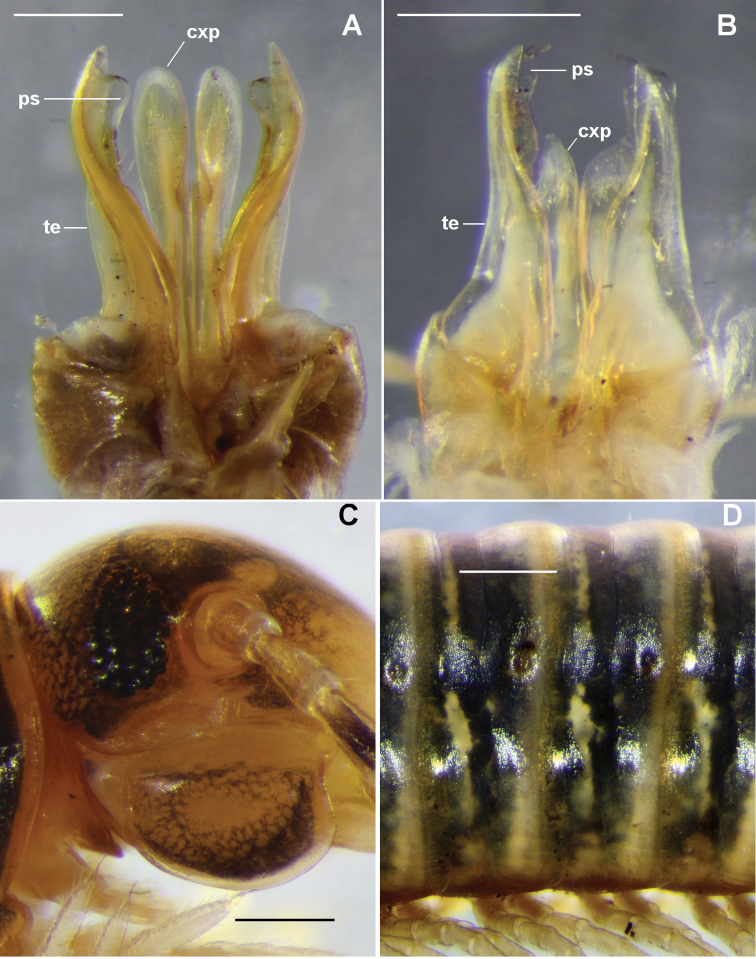
**A** *Amastigogonus tasmanianus* Brölemann, 1913 (type species of *Amastigogonus*), QVM 23:54404 **B–D** *A. insularis* sp. n., holotype, QVM 23: 46411 **A, B** Posterior views of gonopod complex; **C** right lateral view of head **D** left lateral view of midbody rings **cxp** coxite process **ps** pseudoflagellum **te** telopodite. Scale bars = 0.5 mm.

In a previous paper (Mesibov 2017) I reviewed the endemic Tasmanian genus *Amastigogonus* Brölemann, 1913 and added six new species. In all but one of the 10 known *Amastigogonus* species, the telopodite on the anterior gonopod is slightly longer than the coxite process (Fig. 1A). The latter is typically flattened with a slight lateral concavity, and faces the medial side of the telopodite so that the two structures form a “bird’s beak” in which the pseudoflagellum is partly protected. The coxite process in *A. danpicola* Mesibov, 2017 deviates from this pattern in having shallow fossae at the apex and posterobasally.

In this paper I describe a new *Amastigogonus* species in which the coxite process is reduced (Fig. 1B) and does not protect the pseudoflagellum. The new species has so far only been recorded from two small islands off the Tasmanian east coast.

## Materials and methods

**Figure 2.**
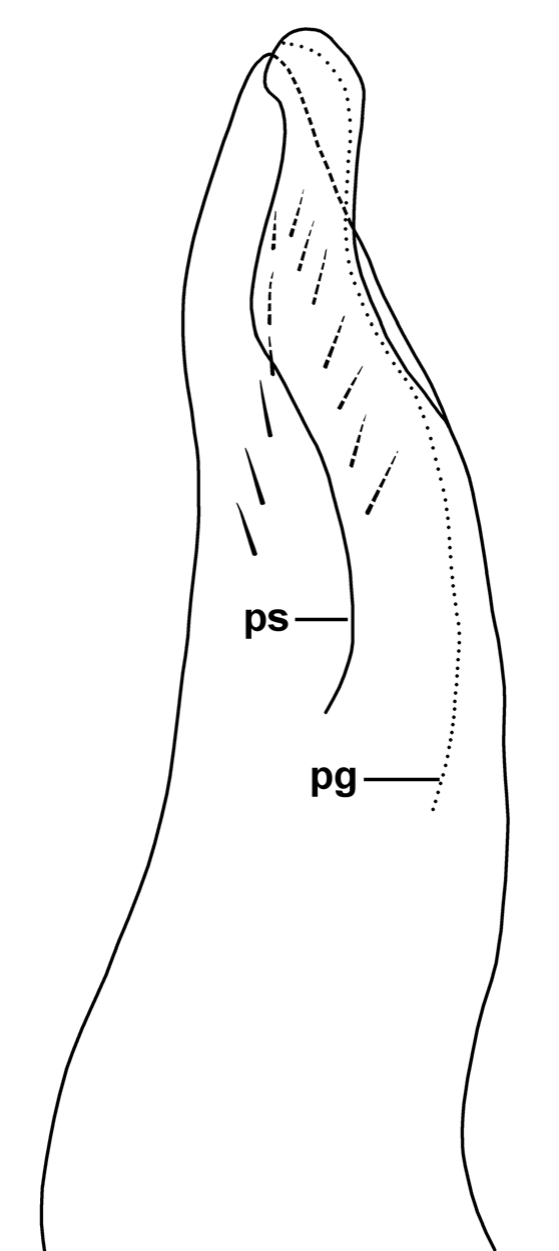
*Amastigogonus insularis* sp. n., paratype from QVM 2017:23:0047. Right anterior gonopod, medial and slightly posterior view of distal portion of telopodite. **pg** prostatic groove **ps** pseudoflagellum. Drawing not to scale.

Specimens are stored in 80% ethanol in the Queen Victoria Museum and Art Gallery, Launceston, Australia (QVM). Photomicrographs in Fig. 1 are focus-stacked composites made with Zerene Stacker 1.04. Photomicrographs were taken with a Canon EOS 1000D digital SLR camera mounted on a Nikon SMZ800 binocular dissecting microscope equipped with a beam splitter. Measurements were made to the nearest 0.1 mm with the same microscope using an eyepiece grid and a reference scale. The gonopod telopodite illustrated in Fig. 2 was temporarily mounted in 1:1 glycerine:water and imaged using an eyepiece video camera mounted on an Amscope binocular microscope. A preliminary drawing was traced from a printed copy of the image, with details confirmed by microscopic examination of the mounted object. Latitude/longitude figures are given in decimal degrees to four decimal places, together with an estimate of spatial uncertainty.

## Results

### Order Spirostreptida Brandt, 1833

#### Suborder Epinannolenidea Chamberlin, 1922

##### Family Iulomorphidae Verhoeff, 1924

###### Amastigogonus insularis sp. n.

Taxon classificationAnimaliaDiplopodaIulomorphidae

http://zoobank.org/1A6BD2B8-CB9D-46E8-AE63-5E653E7DA815

Figs 1B–D , 2

####### Holotype.

Male, Tasman Island, Tasmania, 55G 581536 521818 (GDA94) [-43.2419 148.0042 ±100m], 210m a.s.l., 19 November 2005, S. Bryant + Hamish Saunders Memorial Island Survey Program personnel, pitfall, cliff mosaic, 5A, QVM 23: 46411.

####### Paratypes.

Tasmania: 1 male, 4 females, 1 juvenile, Schouten Island [-42.3131 148.2850 ±2km], 23–26 September 1993, R. Taylor, QVM 2017:23:0047; 4 females, Tasman Island [-43.2384 148.0031 ±500m], 3 June 2016, L. Gadd, 2017:23:0093; 5 females, Tasman Island, -43.2357 147.9996 ±25m, 190m a.s.l., 21 April 2017, R. and L. O’Grady, under she-oak log, QVM 2017:23:0094.

####### Diagnosis.

Male leg 7 coxa not noticeably elongated or distally swollen; coxite process on anterior gonopod much shorter than telopodite, not protecting pseudoflagellum; pseudoflagellum as in *A. peninsulensis* Mesibov, 2017 but longer, extending just past telopodite tip.

####### Description.

As for the genus description (Mesibov 2017, p. 5) with the following details: holotype male with (59+1) rings, 2.0 mm midbody diameter; paratype male with (51+2) rings, 1.6 mm; longest female (in QVM 23:0093) with (63+1) rings, 2.5 mm. Trunk rings dark grey with light annulus posteriorly and irregular, partly annular light markings on prozonite (Fig. 1D); striae on posterior metazonites reaching ca 1/2 ozopore height.

Male with cardo not deeper posteriorly (Fig. 1C). Leg 7 coxa not noticeably elongated or distally swollen. Prefemoral pad ca 1/2 femur length.

Coxite process on anterior gonopod (Fig. 1B) reaching ca 2/3-3/4 telopodite height, not protecting pseudoflagellum. Telopodite (Fig. 2) with slight medial thickening bordered by two rows of short setae behind pseudoflagellum, meeting near telopodite apex. Pseudoflagellum arising at ca 1/2 telopodite height, ca 1/2 telopodite width at base and only slightly narrowing distally; slightly sinuous, curving first posteriorly, then anteriorly, then posteriorly to terminate just distal to telopodite apex, the pseudoflagellum tip bent medially (Fig. 1B); prostatic groove running on anterior edge of pseudoflagellum to posterodistal portion of tip.

####### Distribution.

**Figure 3.**
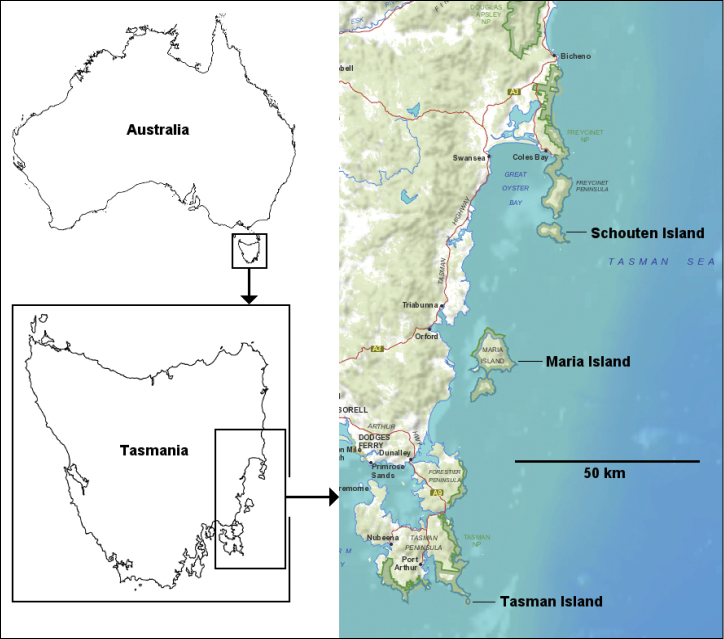
Outline maps of Australia and Tasmania, and topographic map of part of the east coast of Tasmania showing Schouten and Tasman Islands. Mercator projections; topographic basemap from the Land Information Service Tasmania (LIST), copyright State of Tasmania, used with permission.

Schouten and Tasman Islands off the east coast of Tasmania (Fig. 3).

####### Name.

Latin *insularis*, insular; adjective.

####### Remarks.

As with the two known males, the Schouten Island females are smaller than their Tasman Island counterparts: (51+2)–(59+1) rings, 1.5–2.3 mm midbody diameter from Schouten Island, (47+2)–(63+1) rings, 2.3–2.7 mm from Tasman Island. Further, the pseudoflagellum tip in the holotype male from Tasman Island is slightly longer, broader and more medially directed than the tip in the Schouten Island paratype. These are minor differences and I regard the two forms as conspecific.

I doubt that *A. insularis* sp. n. is restricted to its two widely disjunct localities, ca 105 km apart. However, the only iulomorphid so far collected on Forestier and Tasman Peninsulas, just north of Tasman Island (Fig. 3), is *A. peninsulensis* Mesibov, 2017. Similarly, the only iulomorphid recorded from the Freycinet Peninsula (Fig. 3), just north of Schouten Island, is *A. michaelsae* Mesibov, 2017. The latter species is also the only iulomorphid known from Maria Island, halfway between Schouten and Tasman Islands (Fig. 3). I suspect that *A. insularis* sp. n. will be found in future in coastal habitats along the east coast of the Tasmanian mainland, and possibly also on Maria Island.

### Key to males of *Amastigogonus* (see figs 6 and 8 in Mesibov (2017), and Fig. 2 above)

**Table:** 

1	Coxite process of anterior gonopod truncate with apical fossa; cardo deeper in posterior half	***A. danpicola* Mesibov, 2017**
–	Coxite process laminate with broadly rounded apex; cardo deeper in anterior half	**2**
2	Coxite process much shorter than telopodite; no legs with elongated coxae	***A. insularis* sp. n.**
–	Coxite process almost as long as telopodite; elongated coxae on leg 7 only, or on legs 7, 10 and 11	**3**
3	Pseudoflagellum tapering to sharp point	**4**
–	Pseudoflagellum bluntly rounded, expanded or apparently bifid at tip	**7**
4	Pseudoflagellum with distinct anterior shoulder, prostatic groove making sharp S-bend to reach tip; dense field of short, fine setae on telopodite behind pseudoflagellum	***A. fossuliger* Verhoeff, 1944**
–	Pseudoflagellum without anterior shoulder, prostatic groove without S-bend; only sparse, stout setae behind pseudoflagellum	**5**
5	Pseudoflagellum broad at base, abruptly truncate apically, continued as sharp, pointed tip bent over laterally or medially	***A. hellyeri* Mesibov, 2017**
–	Pseudoflagellum not truncate apically	**6**
6	Pseudoflagellum broad at base, gradually tapering to sharp point, not as long as telopodite	***A. hardyi* (Chamberlin, 1920)**
–	Pseudoflagellum narrow at base, very gradually tapering to sharp point, much longer than telopodite	***A. tasmanianus* Brölemann, 1913**
7	Pseudoflagellum with tip strongly curving posterobasally and with small tooth on apicodistal margin, thus appearing bifid	***A. elephas* Mesibov, 2017**
–	Pseudoflagellum with bluntly rounded or expanded tip	**8**
8	Pseudoflagellum with tip strongly curving posterobasally	***A. verreauxii* (Gervais, 1847)**
–	Pseudoflagellum with tip directed distally or slightly posteriorly	**9**
9	Pseudoflagellum with tip slightly expanded apically and truncate, with small tooth medially on distal margin	***A. orientalis* Mesibov, 2017**
–	Pseudoflagellum gradually narrowing to bluntly rounded tip	**10**
10	Pseudoflagellum with tip directed slightly posteriorly and with posterobasal margin extended as bulge	***A. peninsulensis* Mesibov, 2017**
–	Pseudoflagellum with tip directed distally and with prominent sharp tooth on posterobasal margin	***A. michaelsae* Mesibov, 2017**

## Supplementary Material

XML Treatment for Amastigogonus insularis

## References

[B1] Mesibov R (2017) Iulomorphid millipedes (Diplopoda, Spirostreptida, Iulomorphidae) of Tasmania, Australia. ZooKeys 652: 1–36. https://doi.org/10.3897/zookeys.652.12035 10.3897/zookeys.652.12035 PMC5345340 28331389

